# Mobile, Game-Based Training for Myoelectric Prosthesis Control

**DOI:** 10.3389/fbioe.2018.00094

**Published:** 2018-07-11

**Authors:** Brent D. Winslow, Mitchell Ruble, Zachary Huber

**Affiliations:** Design Interactive, Inc., Orlando, FL, United States

**Keywords:** myoelectric prosthesis, amputation, electromyography, pre-prosthetic training, game-based training

## Abstract

Myoelectric prostheses provide upper limb amputees with hand and arm movement control using muscle activity of the residual limb, but require intensive training to effectively operate. The result is that many amputees abandon their prosthesis before mastering control of their device. In the present study, we examine a novel, mobile, game-based approach to myoelectric prosthesis training. Using the non-dominant limb in a group of able-bodied participants to model amputee pre-prosthetic training, a significant improvement in factors underlying successful myoelectric prosthesis use, including muscle control, sequencing, and isolation were observed. Participants also reported high levels of usability, and motivation with the game-based approach to training. Given fiscal or geographic constraints that limit pre-prosthetic amputee care, mobile myosite training, as described in the current study, has the potential to improve rehabilitation success rates by providing myosite training outside of the clinical environment. Future research should include longitudinal studies in amputee populations to evaluate the impact of pre-prosthetic training methods on prosthesis acceptance, wear time, abandonment, functional outcomes, quality of life, and return to work.

## Introduction

There are nearly two million people living with limb loss in the United States, a number that is projected to double by the year 2050 (Ziegler et al., 2008). Major causes of amputation include trauma, peripheral vascular disease, and diabetes (Mcgimpsey and Bradford, 2008). Upper limb amputation in particular is extremely challenging. In addition to physical/functional movements necessary for professional and social activity, the hand is critical for psychosocial roles including gestures, communication, and sensation (Freeland and Psonak, 2007). The resultant changes from the loss of upper limbs to motor and sensory function have the potential to reduce individual independence, quality of life, and employment opportunities by impacting activities of daily living (ADL) such as eating and grooming, using mobile or desktop computers, and coordination.

Myoelectric prostheses provide upper limb movement control using electromyography (EMG) leads on the residual muscles to control arm and hand movements (Dawson et al., 2011). Control mechanisms can vary from a two-state amplitude modulation controller, where EMG signals from a single muscle group control the velocity of a single actuator of the prosthesis (Parker et al., 2006), to dual site activation, where antagonistic muscle groups control increasing device functions, to multi-site activation controls, that leverage additional muscle input sites that have been restored through targeted muscle reinnervation (TMR) procedures or pattern recognition (Oskoei and Hu, 2008). In addition to joint rotation options such as wrist movements, advanced myoelectric prostheses offer substantially more functional capabilities including grip patterns, associated mobile applications, and automatic context-driven changes.

Myoelectric prostheses aide in regaining lost capabilities following upper limb amputation, but require intensive training to effectively operate (Anderson and Bischof, 2014). Training difficulties arise from differences in the motor and sensory signals needed to operate a myoelectric prosthesis as compared to controlling an intact limb, including differences in muscles activated, temporal responsiveness, lack of proprioceptive feedback, and increased weight of the myoelectric device, which require motor learning and cortical remapping (Bongers et al., 2012). Currently most myoelectric training occurs following device arrival, and consists of learning to consciously control muscle contraction, level of activation, and isolation through repetitive exercises (Ison and Artemiadis, 2015). The need to concentrate and continuously react during training is expected to decrease with use, but often takes amputees many months, with the result that many users abandon the prosthesis before mastery is achieved (van der Riet et al., 2013). A recent meta-analysis of self-reported compliance indicates that approximately 30% of patients fitted with myoelectric prostheses eventually abandon the device (Biddiss and Chau, 2007); however the true proportion is likely much higher (Silcox et al., 1993). Difficult pre-prosthetic training has been identified as a primary reason for low user acceptance (Peerdeman et al., 2011).

Available evidence suggests that outcomes are better for users who commence myoelectric prosthesis training following amputation, prior to prosthesis arrival (Romkema et al., 2013). However, current pre-prosthetic training methods and tools are expensive, restricted to inpatient or in-office use (Dromerick et al., 2008), are manufacturer specific, lack training transferability to novel motor tasks (Dawson et al., 2011), and lack personalized training capabilities (Smurr et al., 2009). Another difficulty with current training tools lies in the need to manually apply electrodes to residual limb muscle sites, connect to wired input devices, and maintain connectivity while performing actions to control on-screen simulations, which restricts user range of motion (ROM). Training approaches that use EMG inputs are limited in scope and are not engaging to the user (Terlaak et al., 2015). In addition, current training tools do not collect and report meaningful data to clinicians or therapists.

In the present study, we present a novel, simplified approach to myoelectric prosthesis training, consisting of: a wireless wearable EMG band; a series of prosthetic training games; and a provider website which allows for defining EMG sites, thresholds, and tracking training progress. A non-amputee population was assessed since the number of individuals with a recent transradial amputation who are being provided their first myoelectric prosthesis is low and not large enough for a statistically relevant study (Romkema et al., 2013). In the current study, we hypothesize that participants will significantly improve their ability to control wrist flexor and extensor muscles using their non-dominant limb, including basic activation, muscle sequencing, proportional control and isolation.

## Methods

### Participants

All methods involving participants were approved by a series of Institutional Review Boards (Copernicus Group IRB, Durham NC; US Army Medical Research and Material Command Human Research Protection Office [HRPO], Fort Detrick, MD).

Twelve able-bodied participants (6 male; average age 30.6 ± 6 years) were recruited for the study, which lasted approximately 1 week. Participants were recruited using recruitment flyers posted online and through recruitment fairs at local universities. Inclusion criteria included age >18 years, sufficient neurological and cognitive function to operate a mobile device, and full use and control of upper limbs.

### Materials

The Auto-Diagnostic Adaptive Precision Trainer for Myoelectric Prosthesis Users (ADAPT-MP) system was utilized for the current effort, which consists of: (1) a wearable, wireless EMG band to display user signals and control games (Myo Gesture Control Armband, Thalmic Labs, Kitchener, Ontario, Canada); (2) a series of mobile games that train upper limb amputees to control myoelectric prostheses; and (3) a HIPAA compliant provider website to supply meaningful data to clinicians and therapists.

**Figure 1:**
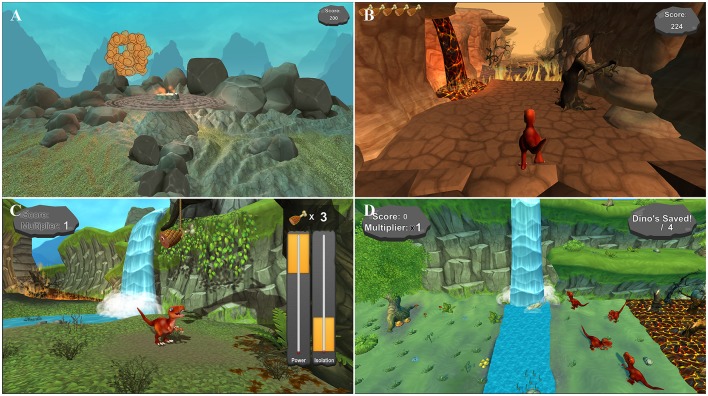
ADAPT-MP mobile games. **(A)** Volcanic Crush teaches basic control, including flexion, extension, and co-contraction. **(B)** Dino Sprint teaches sequences of contractions (flexion, extension, co-contraction). **(C)** Dino Feast teaches muscle isolation (flexor vs. Extensor) and proportionality. **(D)** Dino Claw reinforces isolation and proportionality and allow for practice of activities of daily living via 3-D arm movements.

ADAPT-MP mobile games (Figure 1) train various aspects of myoelectric device control including: basic dual-site muscle activation (Toledo et al., 2010); sequencing (Smurr et al., 2009); proportional control and isolation (Ison and Artemiadis, 2015); 3-D movement control (Bongers et al., 2012); and additionally provide myoelectric signals and visualizations to users (Dupont and Morin, 1994). Games were developed in the Unity platform and function across major operating systems including Android, iOS, and Windows. All games were designed and developed in collaboration with upper limb specialists, including prosthetists and occupational therapists.

Four engaging dinosaur themed games were developed for training purposes: Volcanic Crush (basic dual-site muscle activation), Dino Sprint (sequencing), Dino Feast (proportional control and isolation), and Dino Claw (3-D movement control; Figure 1). Each of the games has 4 levels of difficulty, where higher level difficulties combine more muscle activations/sequences. Game scores are proportional to the number of correct muscle contractions.

Volcanic Crush focuses on “crushing” boulders that erupt from a volcano by activating a particular muscle (flexor, extensor, co-contraction) based on the color the boulders become after a pre-set period of time (2–5 s following eruption). Volcanic Crush was developed to teach basic muscle site control including flexion, extension, and co-contraction, and is based on the psychomotor vigilance task (PVT, Dinges and Powell, 1985). Successfully activating the correct muscle causes the boulder to explode, while failing to perform the correct action or performing no action results in an error. The game ends after five errors are made. Higher game score is associated with an increasing number of correct muscle contractions.

Dino Sprint builds on Volcanic Crush by teaching users sequences of contractions. Users control a dinosaur running an infinite path avoiding obstacles, and must perform certain muscle activations to avoid these obstacles. Failing to perform the correct action, or not performing the correct action in time, results in an error, with five errors as the limit per game. Higher game score is associated with an increasing number of correct muscle contraction sequences.

Dino Feast trains proportional control and isolation, and illustrates the level of user contraction for each muscle site on screen. Contraction thresholds dynamically fluctuate once the user makes a successful contraction. Activating a muscle, or both muscles, within the correct target levels results in a successful contraction, while failing to keep the muscle activations within appropriate thresholds results in an error. Five errors are allowed for each game. Higher game score is associated with an increasing number of isolated muscle flexor contractions without contracting wrist extensor muscles.

The final game, Dino Claw, utilizes the onboard accelerometer to track user limb movement as an additional game input. Similar to ADLs practiced by amputees, users must “pick up” virtual objects of varying sizes, move the objects to new location, and drop them using a combination of limb movement and muscle activation. Dino Claw is a time based game where users are allowed a maximum of 40 s to move a set of targets from the right to the left side of the screen. Game score is proportional to the number of virtual objects moved within the time limit.

### Experimental procedure

Upon arrival, participants provided written, informed consent and were introduced to the ADAPT-MP system, including guided completion of the first level of each game. Participants then completed a series of questionnaires (pre-test) including: demographics; User Evaluation Survey (UES) (Prahm et al., 2017b,c); Intrinsic Motivation Inventory (IMI) (Anderson and Bischof, 2014; Prahm et al., 2017a); and the System Usability Scale (SUS) (Dawson et al., 2012). Participants were then issued the ADAPT-MP system, consisting of the Myo band and Android tablet (NVIDIA shield tablet, Santa Clara, CA, USA), and were instructed to play for 20–30 min daily for 1 week. Participants were instructed to complete levels 1–3 of each game, then to only play level 4 thereafter. Participants were also instructed to use their non-dominant upper limb, since the remaining limb of an amputee always becomes dominant (Prahm et al., 2017c). Following 1 week, participants returned the kit, responded to the UES, IMI, and SUS (post-test), and were debriefed and paid $50 for their participation.

### Measures

The UES consists of 5 questions each using a 5 point Likert scale to evaluate the games in general, the input and control method, and motivation to play (Prahm et al., 2017b,c). Following initial exposure to the 4 training games, participants scored game input, control, motivation and fun using the UES. After 1 week of using the training games, participants scored each game using the UES again. Participants rated training game enjoyment, choice, competence, and immersion using the IMI, previously used in both amputee (Prahm et al., 2017a,b) and non-amputee populations to rate pre-prosthetic training systems (Anderson and Bischof, 2014; Prahm et al., 2017c). The IMI uses a 7 point Likert scale to rate the training system's interest/enjoyment, perceived competence, effort/importance, and tension/pressure. The SUS is a 10-item questionnaire that has been used to evaluate hardware, software, mobile devices, websites and applications (Bangor et al., 2008). Participants respond to various aspects of system usability using a 5 point Likert scale ranging from strongly agree to strongly disagree.

The provider web portal provided quantitative information regarding participant system usage and performance, including: the total amount of training time per session; gameplay information including level, score, errors, and raw or rectified EMG. Maximal rectified EMG was analyzed at both muscle sites prior to and following training.

### Data analysis and statistics

Paired samples *t*-tests were used to evaluate the impact of training on user motivation using the IMI, on game enjoyment using the UES, and on game usability using the SUS, at days 1 (pre) and 7 (post) with α = 0.05. Differences between games were evaluated for the UES using a one-way ANOVA and Tukey *post-hoc* testing with α = 0.05. Differences in amount of time spent playing the training games by day across the week were evaluated using repeated measures ANOVA with α = 0.05.

## Results

The sociodemographic factors for the study sample are listed in Table 1. The average age of the participants was 31 ± 6.0 (SD) years, most were right handed (92%), and most preferred the Apple (Cupertino, CA, United States) operating system (iOS; 83%).

**Table 1:** List of sociodemographic factors in the classifier study sample.

	**Study sample % (n)**
**GENDER**	
Male	50.0(6)
Female	50.0(6)
**AGE GROUP**	
21–25	16.7(2)
25–30	25.0(3)
>30	58.3(7)
**LEVEL OF MOBILE GAMING EXPERIENCE**	
Low	8.3(1)
Moderately Low	41.7(5)
Moderately High	41.7(5)
High	8.3(1)

**Figure 2:**
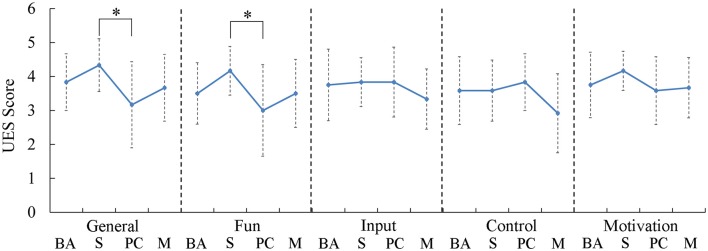
Boxplots of self-reported responses to the user evaluation survey. Participants rated the Dino Sprint (Sequencing) game as significantly higher than Dino Feast (Proportional Control). BA, Basic Activation; S, Sequencing; PC, Proportional Control and Isolation; M, Movement. ^*^p < 0.05.

Users evaluated the games immediately following initial exposure and again following 1 week of training by responding to the UES. No statistical differences were observed pre-post training (Figure 2). However, differences were observed for general user experience [*F*_(3, 44)_ = 2.87, *p* = 0.047] and the degree of fun [*F*_(3, 44)_ = 2.82, *p* = 0.05]. Post-hoc testing indicated both differences were observed between Dino Sprint and Dino Feast for general (*p* = 0.028) and fun (*p* = 0.036).

**Figure 3:**
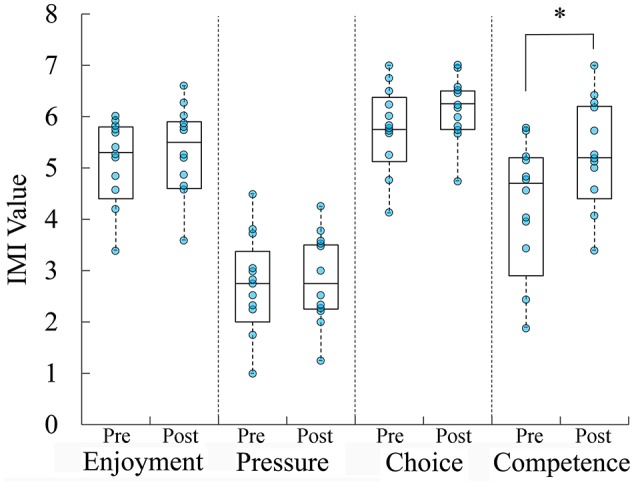
Boxplots overlaid with raw data of self-reported motivation to use the ADAPT-MP system. Participants reported that motivation to use ADAPT-MP significantly increased over the course of the study, due to their increasing competence. ^*^p < 0.05.

Participant motivation increased as a function of training. Total intrinsic motivation significantly increased from baseline [82.0 ± 12.2(SD)] to completion [89.8 ± 8.94(SD); *t*_(11)_ = −2.818; *p* = 0.017]. The increased motivation was a function of increasing participant competence (*p* = 0.026), via analysis of IMI subscales (Figure 3).

**Figure 4:**
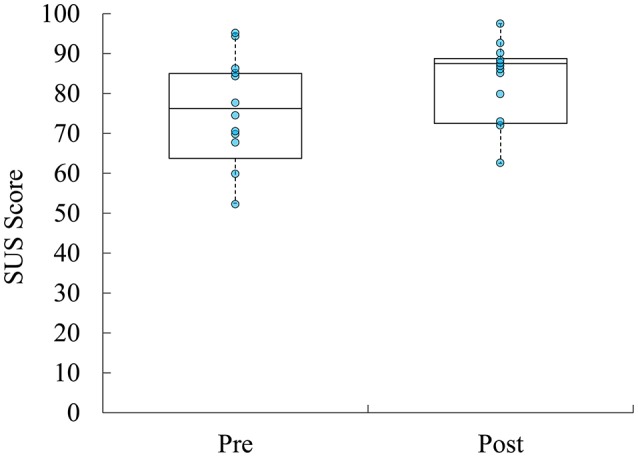
Boxplots overlaid with raw data of self-reported usability of ADAPT-MP system over the course of the study.

Immediately following system exposure, participants rated the training system usability at 75.2 ± 13.2 (SD) using the SUS. Following a week of system exposure, participants rated the training system usability at 83.5 ± 9.91 (SD), which was not statistically significant [*t*_(11)_ = −1.624, *p* = 0.133] (Figure 4).

**Figure 5:**
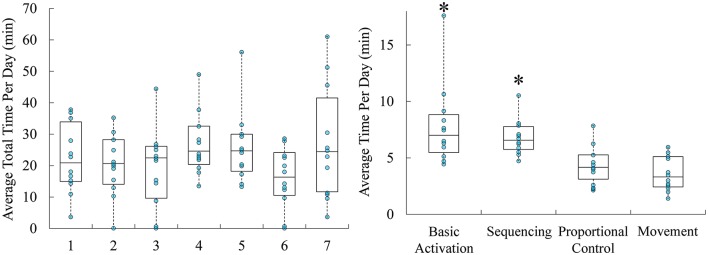
Boxplots overlaid with raw data of average time spent training with the system per day throughout the study (days 1 through 7), and the average amount of time spent on each game per day. Time spend training per day was and average of 22.5 min, with participants spending significantly more time with Volcanic Crush and Dino Sprint. ^*^p < 0.05.

Participants used the training games for an average of 22.5 ± 0.06 (SD) minutes per day. Across the week, there were no differences in the amount of time spent playing daily [*F*_(6, 6)_ = 0.719, *p* = 0.651; Figure 5). However, there were differences in the average amount of time spent playing each game [*F*_(3, 24)_ = 13.547, *p* = 0.000022]. Post-hoc testing indicated that individuals spent significantly more time with Dino Sprint and Volcanic Crush than Dino Feast and Dino Claw (*p* = 0.006).

**Figure 6:**
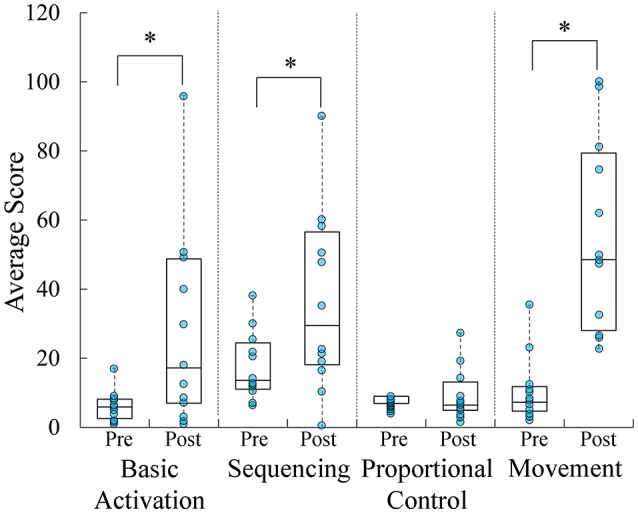
Boxplots overlaid with raw data of average game score throughout the study. Participants significantly improved basic muscle activation, sequencing, and isolation during the course of the study. ^*^p < 0.05.

Between the first and last days of training, participants significantly improved their basic control over flexor and extensor muscle sites as assessed using the Volcanic Crush game [*t*_(11)_ = −2.997; *p* = 0.012; Figure 6). Participants significantly improved their ability to sequence flexor and extensor muscle site activations as assessed using the Dino Sprint game [*t*_(11)_ = −2.868; *p* = 0.015]. Participants did not significantly improve their proportional control as assessed using the Dino Feast game score over the course of the week [*t*_(11)_ = −0.531; *p* = 0.606]. Participants significantly improved their ability to isolate flexor and extensor muscle sites during 3D movements, as assessed using the Dino Claw game [*t*_(11)_ = −6.002; *p* = 0.00009].

**Figure 7:**
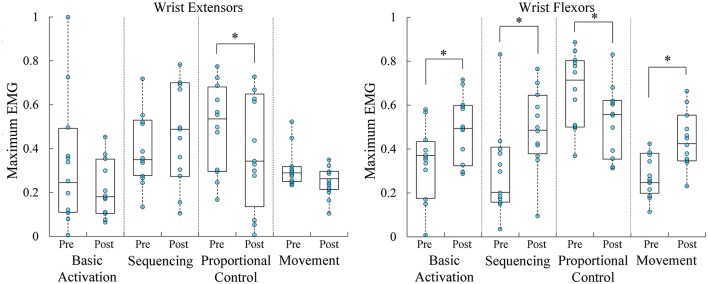
Boxplots overlaid with raw data of maximal rectified EMG, shown at left for wrist extensors and at right for wrist flexors, across the 4 types of training. Wrist flexor maximum EMG increased for basic activation, sequencing, and movement skills. Wrist flexor and extensor maximal EMG decreased during proportional control and isolation. ^*^p < 0.05.

Between the first and last days of training, participants significantly increased their maximal EMG for basic activation of wrist flexors [*t*_(11)_ = −2.352; *p* = 0.038] but not wrist extensors [*t*_(11)_ = 1.256; *p* = 0.235] (Figure 7). Participants significantly increased their maximal EMG for sequencing wrist flexors [*t*_(11)_ = −2.818; *p* = 0.017] but not wrist extensors [*t*_(11)_ = −1.101; *p* = 0.294]. In addition, participants significantly decreased their maximal EMG for both wrist flexors [*t*_(11)_ = 3.197; *p* = 0.009] and wrist extensors [*t*_(11)_ = 2.311; *p* = 0.041] in proportional control and isolation. Finally, participants significantly increased their maximal EMG during whole arm movement of wrist flexors [*t*_(11)_ = −3.345; *p* = 0.007] but not wrist extensors [*t*_(11)_ = 2.145; *p* = 0.055].

## Discussion

The current study indicated that a novel approach to pre-prosthetic training was associated with rapid gains in wrist flexor/extensor control and EMG levels in the non-dominant limb, including muscle sequencing, isolation and movement. The increased control and skill was reflected in the game score, which is associated with an increased number of correct muscle contractions and a reduced number of errors across skills. Participants demonstrated increasing EMG levels across skills with wrist flexors, and increasing proportional control and wrist extensor isolation. Similar results have been described previously using myoelectric prostheses and device simulators (Bouwsema et al., 2014; Clingman and Pidcoe, 2014).

Previous evidence has suggested that poor pre-prosthetic training is closely associated with abandonment of myoelectric prostheses (Peerdeman et al., 2011). For most users, pre-prosthetic occupational therapy following myosite testing focuses on motor training of the chosen muscle sites in absence of visual or other indications that training is progressing (Smurr et al., 2009). For other users, a handful of computer-based training approaches are available during in-office visits, such as MyoBoy and PAULA (Ottobock, Duderstadt, Germany) or Virtu-Limb™ (TouchBionics, Livingston, United Kingdom). However, such computer-based approaches are associated with simplistic graphic representations of rectified EMG, require wired interfaces, and are significantly less motivating and enjoyable than game-based approaches (Prahm et al., 2017b). One group recently showed using the IMI that such computer-based training approaches are significantly less motivating than game-based rehabilitation protocols in upper limb amputees (Prahm et al., 2017b). As compared to popular game format such as maze, racing, or rhythm games used by this group, the ADAPT-MP training games were shown to produce similar levels of self-reported motivation and fun, significantly higher than the previous in-office computer-based training approaches. Participants spent more time, and reported higher levels of general usability and fun with the Volcanic Crush and Dino Sprint games, possibly due to the higher level of difficulty in using proportional control or arm movement as game inputs.

Previous research has indicated that amputees treated within a month of amputation by prosthetists and occupational therapists have a much higher rehabilitation success and return to work rate than those seen later (Bowker, 2004). Currently, prosthetists and occupational therapists lack tools that can be sent home with patients to allow for training between visits (Tabor et al., 2017). Given fiscal and geographic constraints, an additional 20 min of training per day, as observed in the current study, has the potential to improve rehabilitation success rates by providing myosite training outside of the clinical environment. In both amputee and able-bodied samples described previously, the major issues with current approaches to myosite training were identified as: boring and unengaging training (Tabor et al., 2017); training not accessible outside the clinic (Tabor et al., 2017); and training does not provide patient performance metrics to a provider when used remotely by a patient (Dawson et al., 2012). The current system addresses each of these concerns by presenting engaging, mobile training to amputees and is associated with a cloud and web-based approach to data communication for providers.

The EMG band used in the current study is restricted to transradial site locations and adult populations due to the size of the band, and to EMG sites located at the same distance distally on the residual limb. Many amputees associated with polytrauma may not have muscle flexor and extensor sites located at the same distance distally, requiring alternative EMG approaches to training (Smurr et al., 2008). The transferability to an amputee population and the relatively short time-course of the experiment is of concern since the study was conducted with healthy non-amputees over the course of a week. In the current study, the non-dominant limb was used to model upper limb muscle training following amputation (Prahm et al., 2017c), with wrist flexors and extensors expected to be relatively weak and untrained compared to the dominant arm (Nicolay and Walker, 2005). However, following amputation, individuals experience significant reorganization of the central and peripheral nervous systems (Karl et al., 2001), phantom movements and pain (Flor et al., 2006), and utilize different strategies to control a myoelectric device than able-bodied participants. In addition, there is a lack of randomized, controlled clinical data supporting pre-prosthetic training on functional outcomes in amputees (Dromerick et al., 2008). Previously published clinical research on the effect of pre-prosthetic training on amputee health outcomes is represented by small, uncontrolled case studies (Dromerick et al., 2008; Toledo et al., 2010; Resnik et al., 2011; Chadwell et al., 2016; Liu et al., 2016). Future research should include longitudinal studies to evaluate the impact of pre-prosthetic training methods on prosthesis acceptance, wear time, abandonment, functional outcomes (Resnik et al., 2013), quality of life, and return to work. Future work should also include randomized controlled trials to compare new pre-prosthetic training methods to the current standard of care.

## Author contributions

BW and ZH designed the experiments and analyzed the data. BW, MR, and ZH wrote the manuscript.

### Conflict of interest statement

BW, MR, and ZH were employed by company Design Interactive, Inc.
